# Perineural Invasion in Head and Neck Cutaneous Squamous Cell Carcinoma

**DOI:** 10.3390/cancers16213695

**Published:** 2024-11-01

**Authors:** Michelle Pei, Matthew Wiefels, Danielle Harris, Jaylou M. Velez Torres, Carmen Gomez-Fernandez, Jennifer C. Tang, Leonel Hernandez Aya, Stuart E. Samuels, Zoukaa Sargi, Donald Weed, Christine Dinh, Erin R. Kaye

**Affiliations:** 1 Department of Otolaryngology, University of Miami Miller School of Medicine, Miami, FL 33136, USAmxw599@med.miami.edu (M.W.); dweed@med.miami.edu (D.W.);; 2 Department of Pathology and Laboratory Medicine, University of Miami Miller School of Medicine, Miami, FL 33136, USA; 3 Sylvester Comprehensive Cancer Center, Miami, FL 33136, USAssamuels@med.miami.edu (S.E.S.); 4 Department of Dermatology and Cutaneous Surgery, University of Miami Miller School of Medicine, Miami, FL 33136, USA; 5 Department of Medicine, Division of Medical Oncology, University of Miami Miller School of Medicine, Miami, FL 33136, USA; 6 Department of Radiation Oncology, University of Miami Miller School of Medicine, Miami, FL 33136, USA

**Keywords:** perineural invasion, cutaneous squamous cell carcinoma, skin cancer, head, neck

## Abstract

**Background/Objectives:** Cutaneous squamous cell carcinoma (cSCC) is the second most common skin cancer, with a lifetime risk of 14–20% that is rising every year. Although prognosis for cSCC is generally good, certain high-risk features of cSCC portend increased rates of nodal and distant metastasis, recurrence, and disease-specific mortality. One such high-risk factor is perineural invasion (PNI), which is broadly defined as the invasion of cancer into and around nerves. Compared to other high-risk factors, PNI presence is associated with the highest risk for locoregional and distant metastasis. Still, the mechanisms underlying the pathogenesis of PNI remain poorly understood. Recent studies suggest the migration and invasion of tumors into nerves is a result of complex molecular crosstalk within the tumor-nerve microenvironment, wherein the milieu of signaling molecules simultaneously promote neuronal growth and tumor cell invasion. **Methods:** Understanding the molecular and cellular mechanisms that promote PNI will lead to future developments of targeted therapies that may improve locoregional control and survival. **Results/Conclusions:** In our article, we aim to provide a comprehensive review of recent findings about the pathogenesis of PNI, clinical implications of PNI-positive disease in cSCC, available treatment modalities, and potential future therapeutic targets.

## Simple Summary

Cutaneous squamous cell carcinoma (cSCC) is the second most common skin cancer, with the head and neck region being the most common location. Perineural invasion (PNI), or the invasion of cancer into and around nerves, is a high-risk factor in cSCC associated with poor outcomes. The mechanisms of PNI in cSCC are poorly understood, but recent studies suggest that cross-communication between nerve components and cancer is a contributing factor. A better understanding of the mechanisms that promote PNI may lead to new therapies that improve outcomes in cSCC patients.

## 1. Introduction

Cutaneous squamous cell carcinoma (cSCC) is the second most common cutaneous malignancy worldwide, accounting for an estimated 20–50% of all skin cancers [1]. The incidence of cSCC has been steadily increasing over the last several decades [2,3,4], with one recent study finding a 263% increase in age-adjusted incidence of cSCC between 1976–1984 and 2000–2010 [5]. Due to cumulative damage from ultraviolet light, body areas with significant sun exposure, such as the head and neck, are particularly prone to disease development. Cutaneous SCC of the head and neck (HNcSCC) accounts for an estimated 80–90% of all cSCC cases [6].

Cutaneous SCC is a highly curable disease, especially when diagnosed early, with reported 5-year overall survival (OS) rates greater than 90% [7]. However, prognosis is significantly worse when high-risk features are present, such as large tumor size, greater depth of invasion, poor histological differentiation, extensive nodal disease, history of immunosuppression or prior radiation, lymphovascular invasion, or perineural invasion (PNI) [8].

Broadly defined, PNI is the migration and invasion of neoplastic cells from their primary site into, around, and through nearby nerves [9]. PNI is present in an estimated 2–14% of all cSCC cases and often occurs concurrently with other high-risk tumor features such as large tumor size and locoregional metastasis. Clinical studies have consistently demonstrated that PNI is associated with higher rates of locoregional recurrence, nodal metastasis, distant metastasis, and worse disease-specific survival [10,11,12].

Though the prognostic significance of PNI is widely accepted, the molecular mechanisms underlying PNI remain under investigation. Recent studies support the current prevailing theory that PNI results from molecular crosstalk within the tumor-nerve microenvironment between cancer, nerve, and supporting cells. Because of our incomplete understanding of the pathogenesis of PNI, there are sparse treatment options specifically tailored for PNI-positive disease. In this article, we aim to provide a comprehensive review of the latest developments in understanding the pathogenesis underlying PNI, novel observations of the prevalence and clinical endpoints of PNI-positive cSCC, and recent studies on the effectiveness of treatment options. 

## 2. Characteristics of PNI and HNcSCC

### 2.1. Background on PNI and HNcSCC

Among the various anatomical locations where cSCC can arise, head and neck sites are most common, accounting for approximately 60–90% of cSCC cases [13,14]. Because cSCC generally affects regions of the body that are more easily visualized, it is typically diagnosed early and treated successfully. As such, the prognosis of cSCC is generally excellent, with low rates of metastasis and disease-specific death, ranging from 1.2–5% and 2.1–3%, respectively [15,16,17]. However, certain high-risk features of cSCC are associated with advanced disease and worse survival. Patient-related high-risk features include recurrent disease, the presence of neurologic symptoms at the time of diagnosis such as facial nerve palsy or facial numbness, a history of immunosuppression, or prior radiation. Tumor-related high-risk features include large tumor size greater than 2 cm in diameter, depth of invasion greater than 2 mm, poorly defined lesion borders, poor degree of histological differentiation, lymphovascular invasion, and perineural invasion (PNI). Additionally, regardless of tumor size, tumor location in the head and neck, hands, feet, pretibial, or anogenital regions is also considered high risk [18,19,20]. 

The presence of PNI in HNcSCC worsens prognosis, correlating with lower disease-free survival and higher local and regional recurrence [10,21,22]. Extension of PNI into the skull base can have significant neurological consequences, including motor or sensory nerve dysfunction, neuropathic pain, and, if not treated, death [23,24]. 

### 2.2. Pathogenesis of PNI

Malignant cells are characterized by rapid cell division, lack of cellular differentiation, and the ability to migrate away from the primary tumor and spread to distant sites. Metastatic spread can occur via several mechanisms, including direct invasion into surrounding tissues, lymphatic channels to regional lymph nodes, hematogenous spread to other sites in the body, and nerve invasion. While metastatic spread via lymphovascular channels is well studied, spread via PNI has only recently received research attention.

PNI was first described by scientists studying head and neck cancer in the 1800s, who observed cancer spread along cranial nerves (CN) towards the skull base [25]. Historically, PNI was thought to result from an extension of lymphatic metastasis or through the low-resistance pathway of the loose areolar nerve sheaths [25]. Both of these theories have been disproved by later studies that demonstrated minimal lymphatic penetration into nerve sheaths, which are also highly resistant to tumor penetration given their tightly knit composition of multiple layers of collagen and basement membranes [11]. Currently, the prevailing theory of PNI suggests it is a dynamic process involving reciprocal molecular signaling between tumor, neurons, and non-neuronal support cells within the tumor-nerve microenvironment [26]. Though there is a growing body of literature supporting this theory, the exact mechanisms directing tumor invasion into nerves are not yet fully elucidated. 

In the context of PNI, the relationship between nervous system cells and cancer cells is thought to be reciprocal and facilitated by paracrine signaling. Neurons and neuronal support cells release molecules that enhance oncogenesis, while cancer cells release molecules that promote nearby neuritogenesis and angiogenesis [27,28]. It is not completely clear whether tumor invasion of nerve precedes nerve innervation of tumor, or vice versa. Initially, during cancer development, rapid division of malignant cells leads to the development of hypoxic pockets within a tumor, particularly in areas where the rate of neovascularization has yet to catch up to the rate of cell division. Cancer cells react to this hypoxic stress by altering their signaling pathways and releasing a myriad of cytokines and growth factors into the surrounding environment to activate downstream pathways promoting tumor proliferation and migration [29]. 

**Figure 1:**
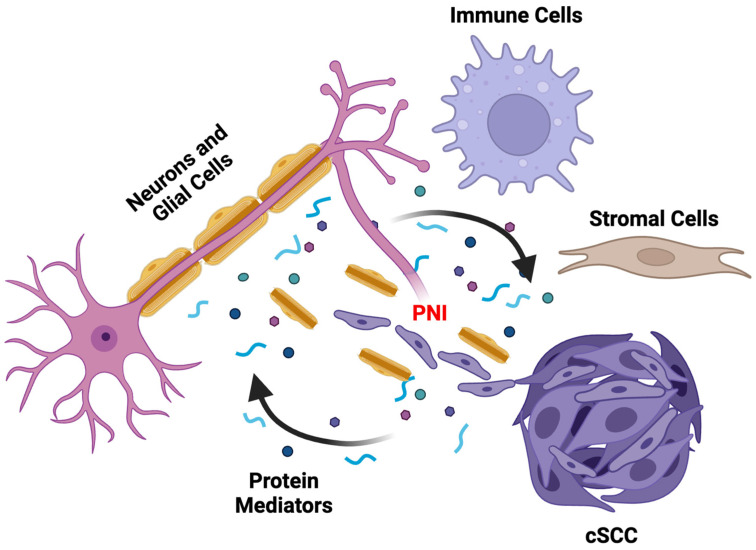
**Generalized Diagram of the Tumor-Nerve Microenvironment and the Pathogenesis of PNI.** Cancer, nerve, and support cells secrete signaling molecules to enhance oncogenesis and neuritogenesis. These mechanisms are thought to be the primary drivers of the development of PNI.

In response to molecular signals from cancer cells, nerves and ancillary cells in the tumor-nerve microenvironment, such as glial cells (e.g., Schwann cells), immune cells (e.g., macrophages), and stromal cells (e.g., fibroblasts), can transform and exhibit altered phenotypes that further enhance cancer proliferation and invasion (Figure 1) [30]. These will be discussed in more detail in the next few paragraphs. 

Nerve cells secrete neurotrophic factors, a family of proteins that are essential in the development, proliferation, and functions of neurons [31]. Neurotrophic factors, including brain-derived neurotrophic factor (BDNF), nerve growth factor (NGF), neurotrophin-3 and -4 (NT-3, NT-4), and glial cell line-derived neurotrophic factor (GDNF), are thought to be important mediators in the PNI disease process, as they have been shown to induce cancer cell growth towards nerves and promote axonal growth of neurons towards cancer. Amongst neurotrophic factors, GDNF is one of the most well-studied in the context of PNI, though most existing studies were performed using pancreatic and gastric cancer models [32]. Research in pancreatic cancer shows consistent overexpression of GDNF by both cancer and nerve cells, which acts through the Ret proto-oncogene (RET) receptor and GDNF family receptor (GFR) α1 to attract cancer cells to nerves [33,34]. In the head and neck, Lin et al. demonstrated in 2017 using an oral SCC model that GDNF upregulates PD-L1 expression, both of which are expressed significantly higher in cancer cells with PNI than those without PNI [35]. The authors also showed the addition of GDNF in a co-culture of DRG with oral SCC cells enhanced PNI. Specific to cSCC, Brugiere et al. found significantly higher expression of neurotrophin BDNF and its receptors TrkB and p75NGFR in perineural tumor cells compared to tumor cells distant from the nerve [36]. To date, no HNcSCC-specific studies exist examining the relationship between neurotrophic factors and PNI. 

Investigations on the mechanisms of PNI development show evidence that Schwann cells and secreted cytokines play an important role in driving PNI. In normal conditions, Schwann cells myelinate nerves during development and repair nerves following injury. During the latter process, myelinating Schwann cells de-differentiate, undergo reprogramming, and differentially express thousands of genes to become repair Schwann cells [26]. Repair Schwann cells secrete trophic factors and cytokines that promote autophagy, neuronal growth, and the formation of nerve regeneration tracks known as Bungner bands [37]. Recent studies have shown that Schwann cell repair mechanisms may be exploited by cancer to facilitate PNI by: (1) releasing neurotrophic factors that induce axonogenesis and accelerate PNI; (2) degrading extracellular matrices to prime the tumor microenvironment (TME) for cancer growth and migration; and (3) providing scaffolding for tumor migration and invasion [38]. Deborde et al. performed in vivo and in vitro studies using pancreatic cancer cells and demonstrated that Schwann cells can organize into tracts and herd cancer cells toward neurites [26]. Furthermore, Schwann cells have been shown to exhibit similar activities in colon cancer and oral SCC in both ex vivo and in vitro studies [39]. Molecules secreted by cancer-activated Schwann cells that have been implicated in PNI include neural cell adhesion molecule 1 (NCAM1), L1 cell adhesion molecule (L1CAM), transforming growth factor β (TGFβ), C-C motif chemokine ligand 2 (CCL2), matrix metalloproteinase (MMP) 2, MMP9 (MMP9), nerve growth factor (NGF), and brain-derived neurotrophic factor (BDNF) [38,40,41,42,43,44,45].

Beyond Schwann cells, other immune and stromal cells have also been shown to influence the interactions between cancer and nerve cells to facilitate the progression of PNI [46]. Tumor-associated macrophages (TAMs) are derived from circular monocytes and differentiated in the presence of cancer. TAMs are divided into the M1 and M2 subtypes; the former exhibits anti-tumor effects, and the latter promotes tumor growth and immunosuppression. Studies in pancreatic cancer show most TAMs in the TME are of the M2 subtype, which are activated by Th2 cytokines and secrete a wide array of cytokines that promote PNI. These include matrix metalloproteinases (MMPs), which degrade extracellular matrices to enable cell migration, pro-inflammatory cytokines interleukin-8 (IL-8) and tumor necrosis factor (TNF), immunosuppressive molecule PD-L1, and neurotrophic factors NGF and GDNF, which directly promote neuritogenesis [47,48,49]. Cancer-associated fibroblasts (CAFs) are stromal cells in the TME that enhance tumorigenesis, angiogenesis, cancer cell proliferation, migration, and invasion via the secretion of various cytokines [46]. Few studies exist examining the relationship between TAMs, CAFs, and HNSCC PNI. Knops et al. found CAFs make up to 80% of tumor volume in late-stage HNSCC tumors, and increased CAF density is associated with tumor spread and metastasis, including perineural invasion [50]. A 2015 retrospective study of 10 PNI-positive HNSCC cases demonstrated high Yes-associated protein (YAP) expression by fibroblasts in areas with PNI, compared to fibroblasts in normal stroma, which suggests YAP may play a role in PNI development [51]. There have been no studies directly examining the relationship between TAMs, CAFs, and the development and progression of PNI in HNcSCC. 

In recent years, there has been growing interest in studying the mechanisms of PNI in HNSCC and HNcSCC. Scanlon et al. co-cultured human HNSCC cells with rat dorsal root ganglia (DRG) and demonstrated that neurons secreted galanin, which activated GALR2 on cancer cells, resulting in prostaglandin E2 secretion to promote neuritogenesis and facilitate PNI [52]. In contrast, Gil et al. co-cultured two HNSCC cell lines (QLL2 and SCC25) with DRG and did not observe cancer invasion of nerves, suggesting certain cancer cell characteristics are necessary for PNI [34]. Until recently, there had been no in vivo models of PNI related to HNcSCC. The first in vivo mouse model of PNI in HNcSCC was described by de Lima et al. in 2023 [53]. In this model, A431 cells (an established cSCC line) were injected into the whisker pads, an area rich in sensory neurons from the trigeminal nerve, of BALB/c Foxn1nu and NSG-A2 mice. Tumors grew in all injected mice, with 1 of 10 (10%) BALB/c Foxn1nu and 2 of 7 (28.6%) NSG-A2 mice developing histologically proven PNI, a rate that is comparable to the reported 2–14% incidence of PNI in human HNcSCC. 

Several molecules have been associated with PNI in patients with HNSCC. Warren et al. showed diffuse overexpression of p53 on immunohistochemistry of HNcSCC with clinical PNI (cPNI) and alterations in signaling molecules known to regulate p53 activity [54]. The authors concluded that p53 gain-of-function mutations may contribute to the development of PNI in HNcSCC. Furthermore, Zheng et al. found upregulation of a pro-angiogenic factor (HIF1A), cell proliferation pathways (MAPK8, mTOR, RAB23), and anti-apoptotic proteins (BCL2L1) were significantly associated with cPNI in HNcSCC [55]. Zilberg et al. found that FGFR2 mutations occurred exclusively in HNcSCC patients with histologically proven PNI [56]. Comparing the gene transcription profiles of 45 patients with HNcSCC, Eviston et al. reported significant differences in mRNA expression of 144 genes between PNI and non-PNI cases, including molecules associated with pathways in extracellular matrix organization, integrin cell surface interactions, insulin-like growth factor (IGF) and IGF binding protein transports, MAPK signaling, and various other cascade pathways [57].

### 2.3. Clinical Presentation of HNcSCC with PNI

PNI has been recently subdivided into two categories: clinical PNI (cPNI) and incidental PNI (iPNI). Patients with cPNI present with radiological evidence and/or clinical signs of nerve invasion by tumor, such as numbness, tingling, pain, or paralysis. In the existing literature, cPNI is also sometimes referred to as perineural spread, perineural tumor spread, or radiologic PNI [11,23]. CPNI in HNcSCC typically involves one or more larger, named CNs and most commonly affects the trigeminal and facial nerves due to their extensive innervation territory in the head and neck [58,59,60,61,62]. Multiplanar, contrast-enhanced magnetic resonance imaging (MRI) is the imaging modality of choice for detecting and diagnosing PNI, with a sensitivity of 95% [63]. Primary imaging features of PNI include contrast enhancement of nerves, widened nerve diameter, and obliteration of perineural fat at foraminal or bony junction openings [64]. Secondary imaging features include unilateral atrophy of muscles secondary to denervation and muscular edema and contrast enhancement of muscle due to increased perfusion [64].

Up to 70% of patients with PNI-positive cSCC, however, present without any clinical symptoms or radiological findings [12,65]. Instead, these patients are diagnosed with PNI-positive disease when PNI is incidentally found on histological examination of their tumor following surgical resection and are thus classified as having iPNI. Comparing cPNI to iPNI, cPNI carries significantly worse locoregional recurrence (37% vs. 17%), disease-specific death (27% vs. 6%), 5-year recurrence-free survival (61% vs. 76%), and disease-specific survival (70% vs. 88%) [12]. 

The trigeminal nerve (CN V) is the largest of the CNs and provides both sensory information from the face, sinuses, nasal, and oral cavity, as well as motor innervation to the muscles of mastication. PNI involving the trigeminal nerve typically manifests as facial numbness, pain, paresthesia, and formication. These sensory symptoms are often subtle and non-specific and may be misdiagnosed as trigeminal neuralgia, delaying cancer diagnosis and treatment [66,67]. PNI can progress, directly invade the trigeminal nerve, and track proximally towards Meckel’s cave, resulting in ocular palsies and conjunctival injection with cavernous sinus involvement. Less common symptoms of CN V PNI include weakened mastication secondary to mandibular nerve involvement [68,69,70].

The facial nerve (CN VII) innervates muscles of facial expression. PNI involvement of the facial nerve presents as a progressive peripheral facial palsy. In the setting of HNcSCC, PNI can involve the facial nerve by direct invasion and/or when the cancer has metastasized to the parotid gland and/or intraparotid lymph nodes. PNI of the facial nerve can be misdiagnosed as a benign peripheral cranial neuropathy such as Bell’s palsy, leading to delays in diagnosis and treatment [71]. A diagnosis of occult malignancy should be considered for patients with facial palsy when there is a history of cutaneous malignancies, immunosuppression, recurrent or bilateral symptoms, insidious onset of facial paralysis, progression after 2 weeks, or lack of recovery after 3 months [72].

Rare presentations of cPNI in HNcSCC include jugular foramen syndrome and leptomeningeal metastasis. Jugular foramen syndrome presents with palsies in CN IX, X, and XI, with clinical symptoms of hoarseness, dysphagia, and pain [73]. Leptomeningeal metastasis is the presence of cancer in the pia and arachnoid mater and manifests as multiple cranial neuropathies [74,75,76,77].

Although only 4% of all HNcSCC patients develop metastasis, the presence of PNI substantially raises this risk [16,22,78,79]. Nodal and distant metastasis have been shown to be associated with large-diameter nerve involvement of tumors greater than 2 cm in diameter [80]. Other risk factors for cSCC metastasis independent of PNI include poor histological differentiation, Breslow thickness >2 mm, and a primary tumor site on the lip or ear [1,12,22]. According to Karia et al., the overall nodal and distant metastatic risk does not significantly vary between iPNI and cPNI; however, further studies are needed [12]. 

### 2.4. Locoregional Recurrence and Survival of Patients with PNI-Positive HNcSCC

In a systematic review of 71 studies, Rowe et al. investigated local recurrence rates in patients with cSCC after various treatments (surgical excision, curettage and electrodesiccation, radiation therapy, cryotherapy, or Mohs micrographic surgery (MMS)) [81]. The average rate of recurrence was 23.3% when follow-up was more than 5 years (Rowe et al.). Recurrence of cSCC has been associated with several tumor characteristics, including tumor size, thickness, PNI, and poor histological differentiation [22]. A meta-analysis of 36 studies by Thompson and colleagues identified Breslow thickness >2 mm as having the highest relative risk of local recurrence (risk ratio [RR] 9.64; 95% confidence interval [CI], 1.30–71.52) [22]. Although the reporting and definition of PNI varied between studies, the authors found PNI to also increase the relative risk for local recurrence (RR, 4.30; 95% CI, 2.80–6.60) [22]. Furthermore, in a systematic review of 12 retrospective studies, Karia et al. investigated the local recurrence rate among 622 patients with PNI and found the local recurrence rate to be higher with cPNI (37%) than iPNI (17%), respectively [12].

PNI is also associated with higher disease-specific mortality in cSCC. Thompson et al. analyzed the disease-specific death associated with PNI in 6 of 36 articles on cSCC. The authors showed that PNI was associated with a greater risk of disease-specific death (risk ratio of 4.06; 95% CI, 3.10–5.32) [22]. In the systematic review by Karia et al., cPNI was associated with a higher risk of disease-specific death (27% vs. 6%; *p* < 0.001) and 5-year disease-specific survival (70% vs. 88%; *p* = 0.002) when compared to iPNI [12]. When focusing solely on HNcSCC, patients with PNI had lower 5-year disease-free survival (DFS) rates than patients without PNI [10,82]. While the extent of 5-year DFS varied between studies (25–80%), all studies noted that the presence of PNI led to an increased likelihood of cSCC recurrence and/or death.

Overall, PNI is an important prognostic factor when assessing HNcSCC. As PNI-positivity was shown to portend poorer outcomes than expected, significant updates in staging cutaneous tumors were made in the eighth edition of the American Joint Committee on Cancer (AJCC-8) published in 2017 [83]. In addition to including guidelines specific for head and neck cSCC, PNI-positive lesions were upstaged from T2 to T3 in the newer edition [84]. In addition to PNI, other factors, including immunosuppression, number of cSCC lesions, and age, have been acknowledged as important prognosticators for HNcSCC patients [85,86]. 

## 3. Treatment of PNI

### 3.1. Current cSCC Treatment Guidelines

Treatment of cSCC is determined by two main factors: the degree of cancer spread (reflected by staging) and the presence or absence of high-risk tumor features [87]. High-risk tumor features include large tumor size, high-risk locations (e.g., head and neck, hands, feet), recurrent disease, history of immunosuppression or prior chemoradiotherapy, aggressive histologic subtypes (e.g., acantholytic, adenosquamous, sarcomatoid), and the presence of perineural or lymphovascular involvement. When present, these high-risk features are associated with increased rates of local recurrence, metastases, and death from disease. The complete set of cSCC treatment guidelines is published and updated yearly by the National Comprehensive Cancer Network (NCCN) (NCCN Guidelines 2023) [88].

Surgical resection is offered to patients with low-risk disease localized to the primary site. If the lesion depth appears to be limited to the dermis, curettage and electrodesiccation (C&E) or shave removal may be offered. However, if the tumor appears to extend beyond the dermis, patients should undergo MMS or standard excision with 4–6 mm clinical margins. Clinical margins should be cleared of disease prior to reconstruction. Patients with positive margins should be further treated with re-excision or radiation therapy. Comparatively, patients with localized disease and high-risk features are offered MMS or standard excision with wide surgical margins, typically between 6–10 mm [89]. 

The NCCN defines very-high-risk cSCC as having a significant risk of nodal metastasis or extensive local recurrence [88]. For these cases, the guidelines recommend radiologic staging and, after multidisciplinary consultation, consideration of neoadjuvant therapy with immunotherapy. For locally advanced cSCC or cSCC that is unresectable or unlikely to benefit from radiotherapy, multidisciplinary discussion and multimodal treatment options should be considered. Surgery is recommended as primary treatment in the form of MMS or WLE with wider surgical margins; neoadjuvant therapy with cemiplimab should be considered after multidisciplinary discussion. For high-risk cSCC in non-surgical candidates, NCCN guidelines recommend the use of definitive radiation therapy with or without systemic therapy (i.e., immunotherapy or chemotherapy). If surgical margins are positive or if margins are negative but there is perineural or direct nerve involvement, the case should be discussed by a multidisciplinary team of surgeons, oncologists, pathologists, and radiologists. Re-resection, systemic therapy, and/or radiation therapy may be offered to patients with positive margins but no PNI, while adjuvant radiation alone is offered for patients with negative margins but PNI-positive disease.

**Figure 2:**
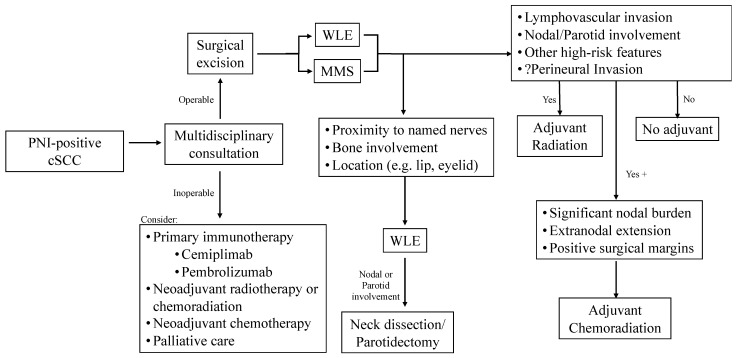
**Simplified Algorithm for Treatment of High Risk cSCC.** PNI: perineural invasion; cSCC: cutaneous squamous cell carcinoma; WLE: wide local excision; MMS: Mohs micrographic surgery.

Cancer spread to regional lymph nodes detected via physical exam or on imaging should be biopsied by fine needle aspiration or core biopsy. Patients with advanced tumors and/or nodal disease should undergo a PET/CT scan or a CT scan of the chest, abdomen, and pelvis with intravenous contrast to rule out distant metastasis prior to treatment. Operable cases should undergo surgical evaluation for resection and neck dissection as clinically indicated. Specifically, neck dissection is recommended in cases of nodal involvement and should be offered in cases of advanced tumors with high-risk features. The NCCN recommends that a primary tumor excision, parotidectomy, and ipsilateral neck dissection be performed for parotid node involvement. The presence of gross (macroscopic) extranodal extension on histologic evaluation warrants adjuvant chemoradiation. Unresectable or inoperable cases, regional recurrence, or metastasis should be referred for multidisciplinary evaluation for radiation therapy, systemic therapy, or both. Patients who decline surgical intervention may be referred to radiation oncology for curative or palliative radiation therapy. A simplified algorithm for the management of high-risk cSCC of the head and neck is presented in Figure 2.

### 3.2. Surgical Treatment Options

MMS is a precise method of removing various localized skin cancers, including basal cell carcinomas (BCCs) and SCCs. This technique is notable for maximizing the preservation of healthy tissue while offering microscopic visualization and control of complete tumor margins. During MMS, thin slices of tissue are removed sequentially and circumferentially deep to and surrounding the clinical margins of a skin tumor. Tissue slices are typically frozen and horizontally sectioned, allowing complete microscopic examination of both peripheral and deep tissue margins. Tissue sections are removed until negative histologic margins are obtained [90]. WLE, or standard excision, is another surgical technique for removing localized skin cancers. For HNcSCC, the tumor is generally excised using 4 to 6 mm gross surgical margins [91].

MMS may be more ideal for certain patients compared to WLE. By preserving more healthy tissue, MMS allows for a smaller surgical defect. Thus, MMS is generally preferred over WLE for cSCC that involves cosmetically sensitive areas like the face. This technique may also be ideal for early-stage cSCC that has not penetrated the deepest layers of the skin [92]. For more aggressive cutaneous head and neck cancers with PNI, a more radical resection with WLE may be favored because these patients may also require radiation, neck dissection, and reconstructive surgery [93]. WLE may be preferred over MMS for cSCC patients with perineural spread to or around the facial nerve because this technique allows for facial nerve monitoring, reducing the risk of damage to the nerve [93,94]. With WLE, a smaller proportion of the excision margin is histologically examined. This limitation can lead to incomplete cSCC excision and an increased risk of recurrence [91,95]. A Dutch retrospective cohort study of 579 patients with 672 HNcSCCs reported an 8% recurrence rate after a 5.7-year median follow-up for patients who received standard excision treatment. In contrast, patients who received MMS had a 3% recurrence rate after a median follow-up of 4.9 years [95]. MMS is also correlated with an increased disease-specific survival rate for cSCC patients compared to cSCC treated with WLE. A multicenter prospective study of 647 patients with 745 cSCCs, 537 of which were HNcSCCs, treated with MMS revealed a 5-year disease-specific survival rate of 99.4% [96].

By definition, patients with iPNI-positive cutaneous cancers have no clinical neurological symptoms on presentation. Early detection of iPNI through histological examination is crucial for favorable disease-free and survival outcomes [97]. In a study of 1177 patients, Leibovitch et al. showed that the presence of PNI in cSCC was correlated with an 8% 5-year recurrence rate compared to a 3.7% 5-year recurrence rate in patients with no PNI (*p* = 0.02) [98]. Compared to WLE without intraoperative margins, tissue cuts from MMS may be better for histological detection of PNI because WLE has a higher chance of producing false-negative margins because of skip lesions, which are areas of tumor cells that may be absent on microscopy because they persist on less visible distal or proximal regions of affected nerves [98,99,100]. PNI depth, defined as the distance from the top of the granular layer or from erosion to the middle of the nerve, is notably shallow in the head and neck, making MMS a reasonable option even in the setting of PNI. A study at our institution by Tang et al. found an average PNI depth of 2.2 mm in HNcSCC, as compared to 4.3 mm in trunk cSCC [101]. PNI-positive cSCC that is not deeply invasive may best be treated surgically with MMS versus standard excision, primarily due to lower cancer recurrence rates with MMS [102]. 

Differences in recurrence and survival rates between MMS and WLE may be observed because the choice of surgery often depends on the stage and location of the cancer. Patients with more invasive cSCC, which is typically treated with WLE, will tend to have worse clinical outcomes regardless of treatment due to an inherently higher risk of metastasis and recurrence. The bulk of the data describing recurrence and survival rates for HNcSCC comes from observational studies, which often do not account for differences in the health of patients, precise tumor location, and cancer stage, all of which may affect treatment choice and prognosis. While MMS may be preferred for earlier stage cSCC that does not involve the facial nerve, WLE with intraoperative margin analysis is often best for treating more invasive tumors that can affect the facial nerve and where other more invasive procedures like neck dissection and/or reconstructive surgery may also be warranted [22,94,103]. Survival outcomes for recently published investigations for cSCC patients with PNI are summarized in Table 1 [10,12,58,62,104,105].

### 3.3. Adjuvant Radiotherapy for PNI

In clinical practice, patients with HNcSCC and high-risk features such as nodal disease, evidence of lymphovascular invasion, and concurrent iPNI are often referred to radiation oncology to receive adjuvant radiotherapy (aRT). aRT may be indicated for patients with cPNI regardless of PNI caliber, given that cPNI patients tend to have significantly worse disease recurrence and survival rates than iPNI patients. aRT may also be considered for patients with iPNI with large caliber PNI (≥0.1 mm), which may indicate more advanced disease that has afflicted larger nerves [12,106]. Large-caliber nerve invasion is the most common indication for aRT for PNI-positive tumors [107]. Carter et al. reported that patients with large caliber PNI (≥0.1 mm) have a higher risk of disease-specific death (HR, 4.5 [95% CI, 1.2–17.0]) and nodal metastasis (HR 5.6 [95% CI, 1.1–27.9]) compared to patients with small caliber PNI (<1 mm) [108]. Though the NCCN guidelines recommend aRT for HNcSCC patients with qualifying nodal metastasis and lymphovascular invasion, it only suggests consideration of aRT for PNI-positive disease (NCCN Guidelines, 2023) [88]. Thus, it is at the clinician’s discretion whether to use aRT for HNcSCC tumors that exhibit this high-risk feature [88,107]. A recent study by Massey et al. included 140 patients with cSCC and found that extensive PNI, defined as invasion of five or more nerves, was a better predictor of prognosis compared to nerve caliber [109]. A study by our own group also identified multiple nerve involvement as an independent poor prognostic factor, indicating that perhaps the number of involved nerves should be incorporated in decision making regarding adjuvant RT [10]. 

There is inconsistent high-quality evidence showing a clear benefit of aRT in the setting of high-risk cSCC, particularly in cases involving PNI [110]. Harris et al. found through a retrospective study of 349 patients with HNcSCC with PNI or regional disease that patients with PNI-positive disease who received aRT had 66% better overall survival (hazard ratio (HR), 0.44; 95% CI, 0.24–0.86) and 63% better disease-free survival (HR, 0.47; 95% CI, 0.23–0.93) compared to patients who received surgical excision alone [32]. A systematic review by Jambusaria-Pahlajani et al., however, reported no significant differences in regional or local metastases, local recurrence rates, or disease-specific death for cSCC with PNI between patients treated with surgery alone versus surgery with aRT [106]. A retrospective study by Ruiz et al. found no difference in outcomes between cSCC patients treated with aRT versus surgery alone [107]. However, this study, which grouped together several outcome measurements including local recurrence, nodal metastasis, and disease-specific death, only included patients with node-negative tumors that had clear histologic margins that, overall, had low baseline levels of recurrence. While some studies control for certain patient demographic characteristics such as sex, age, and tumor characteristics, most existing literature exploring the use of aRT for advanced HNcSCC is limited to observational studies, which often do not account for patient comorbidities, the extent of PNI, surgical margins, and other factors that can affect prognosis [32]. 

There are different tumor characteristics and risk factors that are correlated with poorer outcomes, including the presence of PNI, immunosuppression, thickness, diameter, location, or degree of differentiation. Some risk factors may have more value for predicting poor outcomes. Pêtre et al. found that the total number of risk factors that a cSCC patient had was the strongest predictor of recurrence (HR = 15.110 [95% CI: 3.91–58.40]), with patients having three or more risk factors being at the highest risk for cSCC relapse (*p* < 0.001) [111]. This study also demonstrated that after a number of risk factors and poor tumor differentiation, PNI was the third strongest predictor of recurrence in patients with cSCC (HR = 2.442 [95% CI: 1.11–5.38], *p* = 0.027). aRT may result in better outcomes when a patient has more risk factors for recurrence, metastasis, or death when compared to surgery or radiotherapy alone [107,111]. However, for patients with a more favorable prognosis, especially a lower risk of recurrence, aRT may not yield a significant improvement over surgical monotherapy [107].

The current literature suggests that tumors invading large nerves or multiple nerves may foreshadow worse outcomes that may warrant aRT [112]. A retrospective study of 74 patients with surgically treated advanced HNcSCC by Kampel et al. found that PNI, compared to other tumor characteristics, was associated with the worst overall survival and that patients who received aRT had better overall and disease-free survival compared to patients who received surgical monotherapy [113]. In general, it is reasonable to deduce that aRT should be more strongly considered for cSCC patients with more high-risk characteristics that may portend poorer outcomes. Randomized studies are needed to examine the utility of other monotherapies and combinations of therapies to more fully explore these treatment options.

Some cSCC patients with PNI or other high-risk features may not qualify for surgery. These patients may have preexisting comorbidities such as advanced age, cardiac arrhythmias, or chronic pulmonary disease that may put them at a higher risk for complications from anesthesia or surgery. There are others who would be unlikely to benefit from surgery with or without aRT, including those with terminal disease. These patients should be evaluated on a case-by-case basis by specialist, experienced clinicians. Treatment options for patients with advanced inoperable HNcSCC include palliative care, primary radiotherapy, or primary immunotherapy [114,115].

### 3.4. Systemic Therapies for PNI

There are very few studies examining the use of adjuvant chemotherapy for HNcSCC with PNI, and no randomized controlled trials that directly examine the effect of adjuvant chemotherapy in combination with radiation on PNI. A retrospective cohort study of 61 patients by Tanvetyanon et al. found that patients with high-risk HNcSCC (e.g., high-risk T classification, positive margins, involvement of 2+ nodes, PNI-positivity) who received adjuvant platinum-based chemoradiation had better recurrence-free survival (HR, 0.31; *p* = 0.01) compared to those who received aRT alone, although there was no significant difference in overall survival between the two treatment groups [116]. A clinical trial by Cooper et al. involving 459 patients reported that disease-free survival was significantly higher for those treated with adjuvant cisplatin-based chemoradiation versus aRT alone (HR for disease or death, 0.78; *p* = 0.04). The combined-therapy group in this clinical trial also showed a higher rate of local and regional control (82%) compared to that of the aRT group (72%) after two years [117]. Consistent with the Tanvetyanon et al. study, Cooper et al. found no significant difference in overall survival between the chemoradiation and aRT groups [116,117]. More work investigating the utility of other chemotherapeutic agents combined with aRT is needed to better evaluate and improve the efficacy of adjuvant chemoradiation.

More recent studies have examined the utility of immunotherapy for the management of cSCC with PNI. One retrospective study evaluating the efficacy of immune checkpoint inhibitors for patients with HNcSCC with cPNI found that 82% of patients examined had radiographic evidence of improved perineural disease while on the programmed cell death protein 1 (PD-1) inhibitors cemiplimab or pembrolizumab [118]. Cemiplimab and pembrolizumab are monoclonal antibodies that target the PD-1 receptor on T cells, preventing the binding and activation of PD-1 by its ligand programmed death ligand 1 (PD-L1). By blocking this ligand binding, these monoclonal antibodies help activate T cell-mediated immune responses against cancer cells [119]. Pembrolizumab and cemiplimab are currently the only two Food and Drug Administration-approved immunotherapies for treating patients with advanced cSCC who are not surgical or radiotherapy candidates [114]. 

Another retrospective investigation studying immunotherapy in HNcSCC patients with CN involvement found that administration of primary immunotherapy alone, predominantly cemiplimab, demonstrated clinical response, described as complete response (i.e., complete disappearance of tumor lesions), partial response (i.e., partial disappearance or shrinkage of tumor lesions), or stable disease (i.e., no enlargement or shrinkage of tumor lesions) in 83% of patients after a median follow-up of 23 months (Lopetegui-Lia et al.). The authors also observed that the immunotherapies were well-tolerated with minimal adverse effects; most reported side effects could be managed well with oral medications and did not lead to immunotherapy discontinuation [120]. The efficacy and favorable toxicity profile of cemiplimab suggest that it may be therapeutically useful in the long-term management of cSCC patients with PNI, but further prospective studies examining the efficacy and duration of treatment for this medication and other immunotherapies for these high-risk patients are needed.

There is presently no standard of care regarding adjuvant systemic therapies like chemotherapy or immunotherapy for patients with cSCC with PNI. Currently, there are two ongoing phase 3 adjuvant trials investigating the use of adjuvant immunotherapy in patients with high-risk cSCC (National Clinical Trial (NCT): NCT03969004, NCT03833167). More evidence is needed to provide recommendations for the appropriate duration of immunotherapy and its use in combination with radiotherapy, surgery, or other systemic therapies [114]. Ideally, all patients with cSCC with PNI or other poor prognostic factors should be evaluated by a multidisciplinary team including radiation oncologists, head and neck surgeons, surgical oncologists, Mohs surgeons, and dermatologists [121,122]. Patients should also be involved in multidisciplinary team discussions and their preferences considered when deciding the best course of action.

## 4. Conclusions and Future Directions

cSCC is a common cutaneous malignancy that frequently occurs in the sun-exposed head and neck region. Though the overall prognosis for cSCC is generally good, the presence of PNI is associated with worse outcomes, including higher rates of nodal and distant metastasis, local recurrence, and disease-specific mortality. PNI is believed to result from reciprocal molecular signaling between cancer and neuronal support cells. In response to cancer-secreted cytokines, Schwann cells enhance the development and progression of PNI by increasing axonogenesis, degrading existing extracellular matrices, and organizing and directing cancer cell migration towards neurites. Though significant strides have been made in the pancreatic and colorectal cancer literature to elucidate mechanisms of PNI, few studies exist in HNcSCC [123]. Utilizing what is currently known in other cancer types can provide a framework for future PNI-related studies in HNcSCC. Further investigation may uncover novel therapeutic targets to improve outcomes for patients with advanced-stage HNcSCC.

Currently, treatment for resectable PNI-positive cSCC includes surgical resection and potential adjuvant therapies. Inoperable disease is approached with chemotherapy, immunotherapy, and/or radiation. Immunotherapy is a new, promising treatment modality for PNI-positive cSCC; however, the optimal duration of treatment and its utility in the setting of surgery, radiotherapy, and other systemic therapies are unknown. Future clinical investigations evaluating the impact of immunotherapy alone or in combination with other treatment modalities on locoregional recurrence, nodal and distant metastasis, and survival in advanced-stage PNI-positive cSCC of the head and neck are needed.

## Figures and Tables

**Table 1:** **Survival Outcomes in Cutaneous Squamous Cell Carcinoma (cSCC) Patients with PNI.** A table with recently published studies of survival outcomes in patients diagnosed with cSCC and PNI. cPNI: clinical perineural invasion; iPNI: histological PNI; aRT: adjuvant radiation therapy; CRT: chemoradiation; PFS: progression-free survival; DFS: disease-free survival; HR: hazard ratio; OS: overall survival; CN: cranial nerve; * indicates statistical significance.

Study	Study Type & Patient Details	Treatment	Recurrence	PFS/DFS	OS	Notes
Hazim et al., 2021 [48]	Retrospective study of 21 patients (14 male) with cSCC where cPNI was the presenting symptom 19/21 (90%) had prior excision/Mohs (n = 10) or excision/Mohs with aRT (n = 3)	All with RT 14 with concurrent systemic therapy	10/21 (47.6%)	Median PFS: 21.5 months 2-year PFS: 44.5%	OS at 2 years: 81%	
Cohen et al., 2022 [10]	Retrospective cohort study of 104 patients (87 male) with HNcSCC 61 (58.7%) with PNI; of these, 23 (37.7%) had cPNI and 38 (62.3%) with iPNI	All with surgical resection 45 (43.3%) with aRT 38 (36.5%) with CRT		All PNI: DFS HR 0.33 * cPNI: DFS HR 3.49 * iPNI: DFS HR 1.02	All PNI: OS HR 0.69 cPNI: OS HR 1.79 iPNI: OS HR 0.85	No significant difference in PNI between primary and recurrent tumors
Karia et al., 2017 [12]	Systematic review of 12 studies with >5 cSCC patients with cPNI or iPNI 241 patients with cPNI 381 patients with iPNI	cPNI: 141 surgery+aRT, 34 RT only, 11 CRT, 7 other, 4 surgery alone iPNI: 233 surgery+aRT, 83 surgery only, 42 Mohs+aRT, 15 Mohs only, 10 RT only	cPNI: 37% iPNI: 17% *	Recurrence-free survival at 5 years: 61% in cPNI 76% in iPNI * Disease-specific survival at 5 years: 70% in cPNI 88% in iPNI *	OS at 5 years: 66% in cPNI 43% in iPNI	cPNI: 171 (69.5%) involved CN V 70 (28.5%) involved CN VII 2 (0.8%) involved CN VIII
Erkan et al., 2017 [91]	Retrospective chart review of 21 patients (15 male) with HNcSCC with cPNI 7 (33%) with prior RT	All with surgical resection 14 (67%) with aRT	3 (14%) in 1st year if prior RT	Prior treatment: 1-year DFS: 72% 3-year DFS: 28% No prior treatment: 1-year DFS 91% 3-year DFS 67%	Median OS All: 36 months Previously untreated: 41 months Previously treated: 24 months	Recurrence correlated with positive microscopic margin, poorer DFS, poorer OS
Phung et al., 2022 [52]	Retrospective chart review of 45 patients (32 male) with PNI+ HNcSCC	33 (73%) with surgery+aRT 8 (17.8%) with RT		Median DFS: 1.7 years 5-year DFS: 25%	Median OS: 4.5 years 5-year OS: 45%	Patients with surgery+aRT and negative nerve margins had better DFS *
Schachtel et al., 2022 [92]	Retrospective review of 78 HNcSCC patients (90.4% male) with clinical or histological evidence of PNI in CN VII 89% with recurrent disease 67.1% with CN V involvement	55 for curative intent: 48 with surgery ± aRT 7 with RT alone		Curative intent: 2-year DFS: 66.7% 5-year DFS: 50.7%	Curative intent: 2-year OS: 77.1% 5-year OS: 58.1% All patients: 2-year OS 65.9% 5-year OS 47.5%	
